# The role of mRNA and protein stability in the function of coupled positive and negative feedback systems in eukaryotic cells

**DOI:** 10.1038/srep13910

**Published:** 2015-09-14

**Authors:** Kristian Moss Bendtsen, Mogens H. Jensen, Sandeep Krishna, Szabolcs Semsey

**Affiliations:** 1 University of Copenhagen, Niels Bohr Institute, Blegdamsvej 17, DK-2100 Copenhagen, Denmark; 2 Simons Centre for the Study of Living Machines, National Center for Biological Sciences, GKVK Campus, Bellary Road, Bangalore 560065, India

## Abstract

Oscillators and switches are important elements of regulation in biological systems. These are composed of coupling negative feedback loops, which cause oscillations when delayed, and positive feedback loops, which lead to memory formation. Here, we examine the behavior of a coupled feedback system, the Negative Autoregulated Frustrated bistability motif (NAF). This motif is a combination of two previously explored motifs, the frustrated bistability motif (FBM) and the negative auto regulation motif (NAR), which both can produce oscillations. The NAF motif was previously suggested to govern long term memory formation in animals, and was used as a synthetic oscillator in bacteria. We build a mathematical model to analyze the dynamics of the NAF motif. We show analytically that the NAF motif requires an asymmetry in the strengths of activation and repression links in order to produce oscillations. We show that the effect of time delays in eukaryotic cells, originating from mRNA export and protein import, are negligible in this system. Based on the reported protein and mRNA half-lives in eukaryotic cells, we find that even though the NAF motif possesses the ability for oscillations, it mostly promotes constant protein expression at the biologically relevant parameter regimes.

Many cellular functions, such as regulation of metabolism, decision making, memory, biological rhythm, and homeostasis are emerging from combinations of feedback loops^1^. Positive feedback typically promotes bi- or multistability^2^, allowing cells to be in two or more states (e.g. make a set of genes silent or active). Negative feedback is widely used to induce constant protein expression, however, with a time delay it can result in stable oscillations^3^. In intracellular molecular networks feedback loops are often composed of molecules of different characteristics, i.e. nucleic acids, proteins, and small molecules, and are connected in different ways to perform complex functions and dynamics^4^,^5^. The function and dynamics of these circuits depend on the sign of the feedback loops involved, and on the timescale of the processes constituting the feedback loops. One of the simplest circuits composed of feedback loops is positive autoregulation coupled to a negative feedback, which is a core motif in many cellular pathways both in prokaryotes and eukaryotes^6^,^7^,^8^,^9^. The dynamics of such motifs has been explored using minimal mathematical models^6^,^7^,^8^,^9^,^10^. In these simulations the circuits could produce oscillatory expression of components at a wide range of parameters, and allowed engineering of the characteristics of oscillations. These minimal models assumed a homogeneous, single-compartment intracellular space and operated with a single step protein production process. These assumptions are generally used in models of prokaryotic transcriptional regulation, where transcription and translation occur in the same space and mRNA half-lives are typically short compared to cell division time^11^. However, a recent study demonstrated that in mammalian cells mRNAs are typically long lived, with a median half-life of 9 hours^12^. Also, mRNA production and protein production are separated in space. The time required for processing of mRNAs and for the transport of the processed mRNAs to the ribosomes results in a delay in gene regulation^13^. In order to understand the effect of the time delay and the long mRNA half life, we constructed a more detailed mathematical model for the NAF motif, a circuit composed of coupled positive and negative feedback loops (Fig. 1). We explore the circuit’s dynamics using the experimentally measured ranges of mRNA and protein half-lives. We find that the circuit produces sustained oscillations only in a limited parameter regime where both the activator mRNA and activator protein are short lived. However, the vast majority of transcription factors do not meet these requirements.

## Methods

### Model

**Figure 1:**
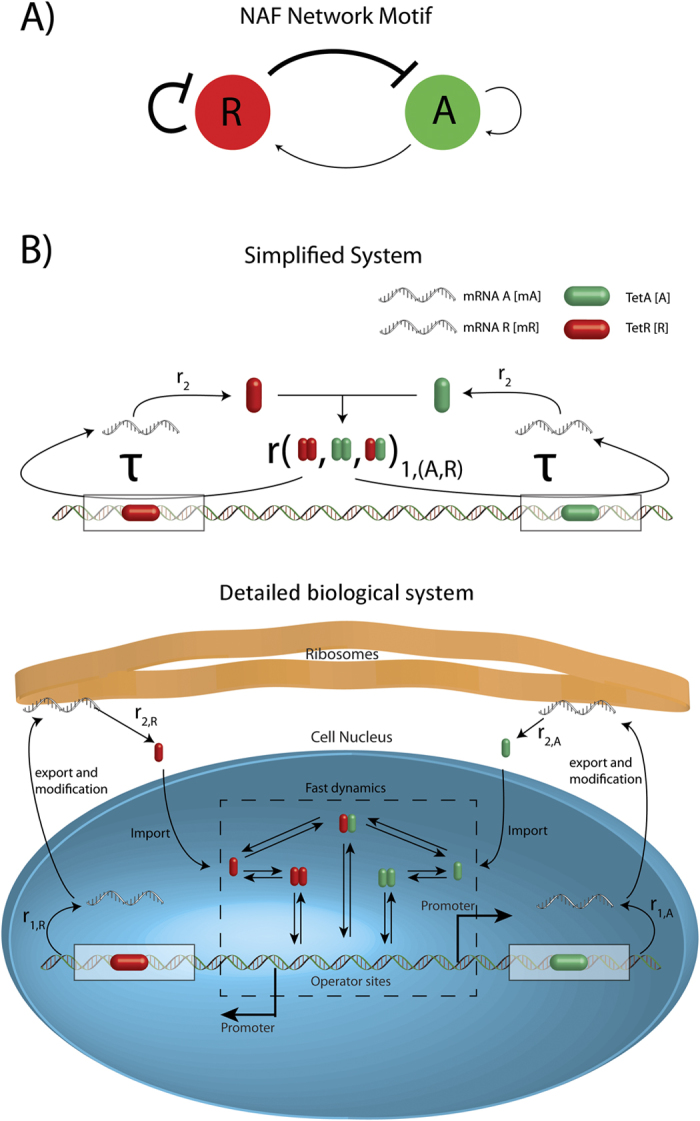
Schematic overview of the model. (**A**) The NAF motif consists of an autoregulated repressor coupled with an autoregulated activator. (**B**) Comparing the biologically realistic system where several steps are involved, We simplify the system as follows. Starting from the top left, mRNA at ribosomes are translated into proteins with rate *r*_2_, the import of proteins is very fast, and therefore it is neglected^19^. Second, the chemical reactions are fast and both dimerization and protein-DNA binding are therefore treated as being in an equilibrium^20^. The last step in the simple model is production of mRNA. This step covers transcription of mRNA, modification and export of mRNA from the nucleus to the ribosomes. The transcription rate is regulated by dimers (activator-activator, repressor-repressor, activator-repressor) binding to the operator sites and the modification and export of mRNA is simplified by a 30 minute time delay (*τ*)^13^,^18^. The simplified system can be schematically represented by the NAF motif in (**A**). The equations governing the simplified system are described in the Materials and methods section.

Positive and negative feedback loops are coupled in different ways in eukaryotic systems^4^. We model a simple situation where an activator (*A*) and repressor (*R*) share common binding sites in the regulation of their own and in each others synthesis. This motif, which is composed of coupled positive and negative feedback and autoregulation (NAF), was previously described in the regulation of long-term memory formation by cyclic AMP (cAMP)-response element-binding proteins (CREB)^8^, and was constructed synthetically in *E.coli*^14^. In a eukaryotic system it can also be easily implemented using the available building blocks of the synthetic Tetracycline Inducible Expression System (TET) (Fig. 1). Most of the interactions between the TET system components have been characterized and the dynamics of various TET-based synthetic networks have been simulated recently^15^. Regulators of the TET system have similar modular structures as eukaryotic regulators, that is, they possess separate DNA binding, dimerization (both from the prokaryotic tetracycline repressor), and regulatory domains. The regulatory domains can either enhance^16^ or inhibit^17^ transcription of promoters located nearby the DNA bound regulatory proteins. For simplicity we model a compact system where the activator and repressor proteins are expressed from two divergent promoters. The expressed proteins bind a common set of operator sites located between the promoters and regulate both their own and each others expression. The dynamics of the system is given by the equations:


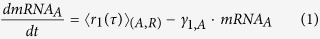



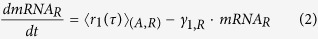



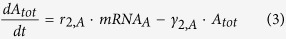



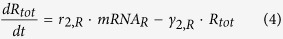


where, *mRNA*_*A,R*_ are the activator and repressor mRNA levels, and *A*_*tot*_, *R*_*tot*_ are the total amount of activator and repressor. *r*_2,*A*_ = *r*_2,*R*_ are the translation rates (assumed equal for activator and repressor), *γ* is the degradation rate and 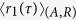 is the transcription rate which is a non-linear function of the repressor *R*_*tot*_ and the activator *A*_*tot*_. *τ* represents the time delay between production of mRNA and its modification and export^13^,^18^. We choose not to include a similar delay in the protein equations because protein import is very fast^19^. The functional form of 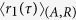 is derived in supplementary (section *Deriving the transcription rate* 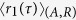) based on the following assumptions:

1. Binding/unbinding of transcription factors to operator sites occurs on timescale much faster than other processes^20^ and is therefore assumed to be in quasi-equilibrium.
2. There is no cooperativity in binding to operator sites^21^.
3. RNA polymerase (and Ribosome) levels do not become a limiting factor even at high expression rates^22^.

### Classifying dynamical behaviour

In practice, in numerical simulations if we observe a minimum of 10 oscillations (i.e. distinct peaks) in the concentration of the repressor with a 2 fold amplitude, we define it as sustained oscillations. If we see less than 10 oscillations with a two-fold amplitude, we define it as damped oscillations. Finally, if we do not observe any peaks in the dynamics of repressor, then we define it as non-oscillatory. Details of how we detect peaks are provided in the supplementary data section F.

### Parameters

The applied transcriptional rates, translational rates, mRNA and protein half-lives are based on a previously reported measurements for about 5000 mammalian genes^12^ (Table 1). Dissociation constants for dimerization and for Protein-DNA binding were based on previous estimates^15^. We assumed a maximum of 100-fold decrease (or increase) in transcription for saturating levels of repressor (or activator)^16^. We were not able to find any experimental data for the half-life of TetR or mRNA of TetR, and previous estimates vary from 10 minutes^15^ to 5 hours^23^.

## Results and Discussion

### Oscillations cannot occur in the completely symmetric NAF system

**Figure 2:**
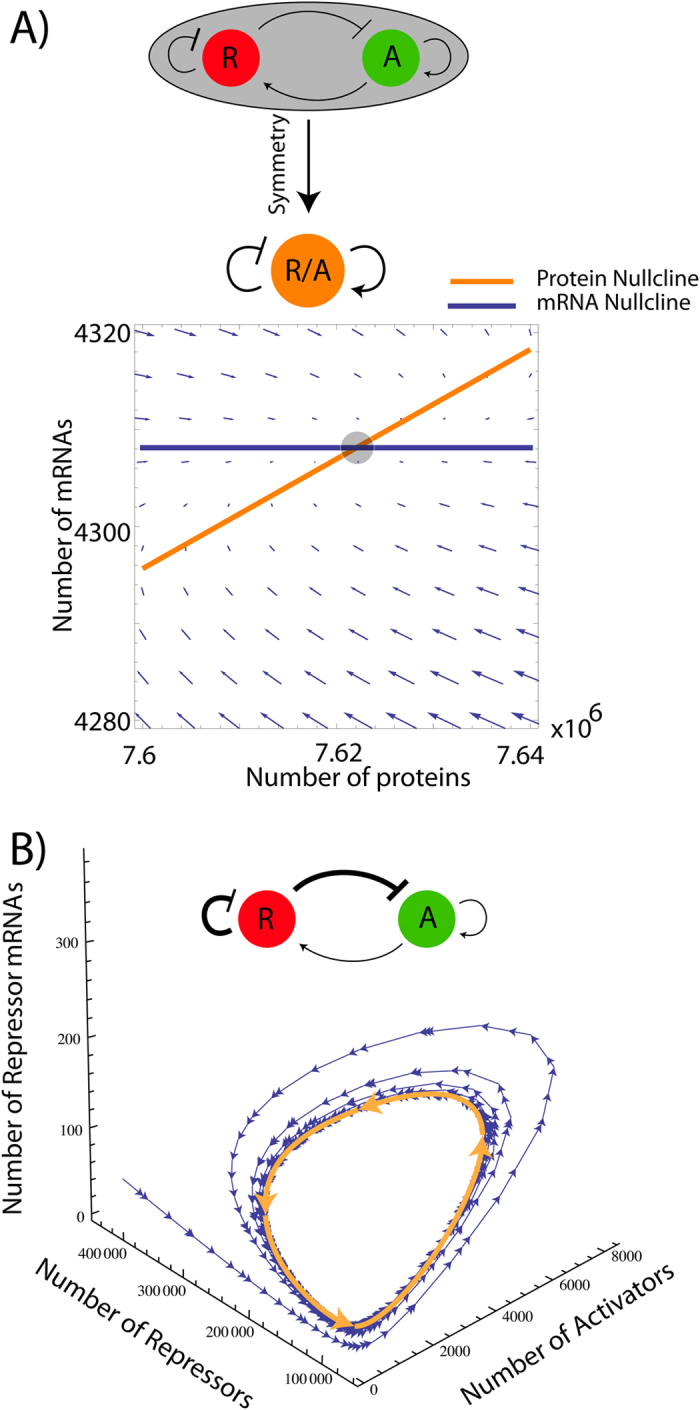
Dynamics of the coupled NAF system both with symmetrical and asymmetric degradations: (**A**) The Tet two-dimensional symmetric NAF motif, where the repressor and activator are collapsed into one protein, cannot oscillate. The system will always settle at a stable node. This two-dimensional system will have the same qualitative behavior as a four-dimensional system where the activator/repressor parameters are identical. (**B**) When the mRNA and protein half-life of the activator is reduced by a factor 10 (to 0.9 hours) the system undergoes a Hopf bifurcation and a stable limit cycle occurs. We show the transient behaviour which settles at the limit cycle, which is highlighted in orange.

When all rates and binding constants that characterize activation are the same as those for repression, and the initial conditions are also chosen to be the same, the system becomes symmetric and reduces to two-dimensions. For such a symmetric system we can prove, using Dulac’s criterion, that there are no limit cycles (see supplementary section *Dulac’s criterion*). This is in agreement with our numerical simulations, (see Fig. 2A).

### Unstable activator and mRNA are required for sustained oscillations

**Figure 3:**
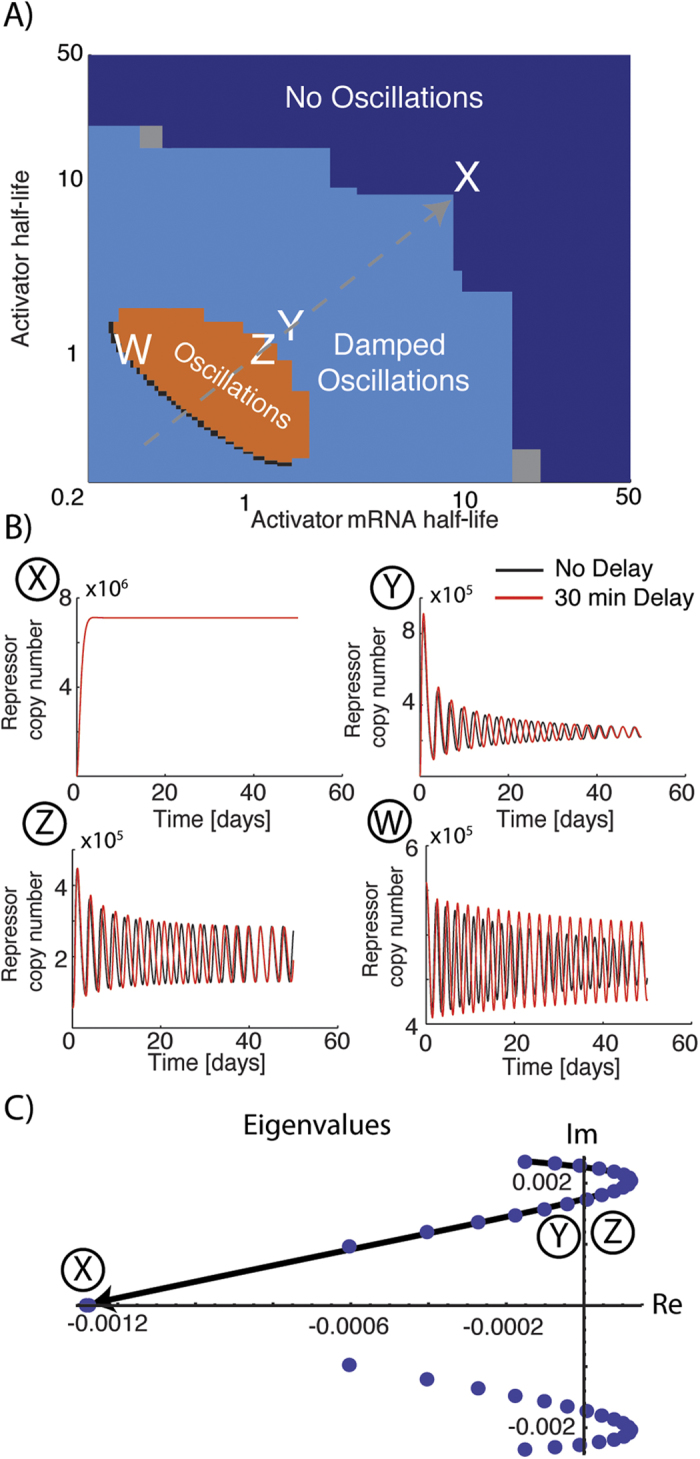
Dynamics of the coupled NAF system: (**A**) A parameter scan of the asymmetry where the repressor protein and mRNA half-lives are fixed at 9 hours and the activator protein and mRNA half-lives are varied from 12 minutes to 50 hours. The effect of a 30 minute time delay is negliglible, since only in a very limited range of parameters the delay actually changes the behaviour (**W**). (**B**) (**X**) For the symmetrical case, we find no oscillations as expected from Fig. 2. We also find that the delay influences the period of oscillation, increasing it by about 10%. (**C**) The transition to oscillations is a Hopf bifurcation, since the real value of the eigenvalues continuously cross the imaginary-axis. There is good agreement between the linearized analysis (**C**) and the simulations (**A**).

If the half-life of the repressor (and its mRNA) is different from that of the activator (and its mRNA), the system can go through a Hopf bifurcation creating a limit cycle (see Figs. 2B and 3C). The half-life range which produces oscillations for the NAF motif is roughly from half an hour to one and a half hour, both for the activator and its mRNA. Note that for half-lives lower than half an hour the motif does not produce sustained oscillations, which is interesting since this means that the activator is needed for sustained oscillations. In other words a pure NAR motif with dimer repression would not produce oscillations in this parameter range. We find agreement between the linear stability of the fixed points and the numerical simulation, where the damping coefficient classification is used (Fig. 3A,C).

### Intermediate repressors stability needed for oscillations

**Figure 4:**
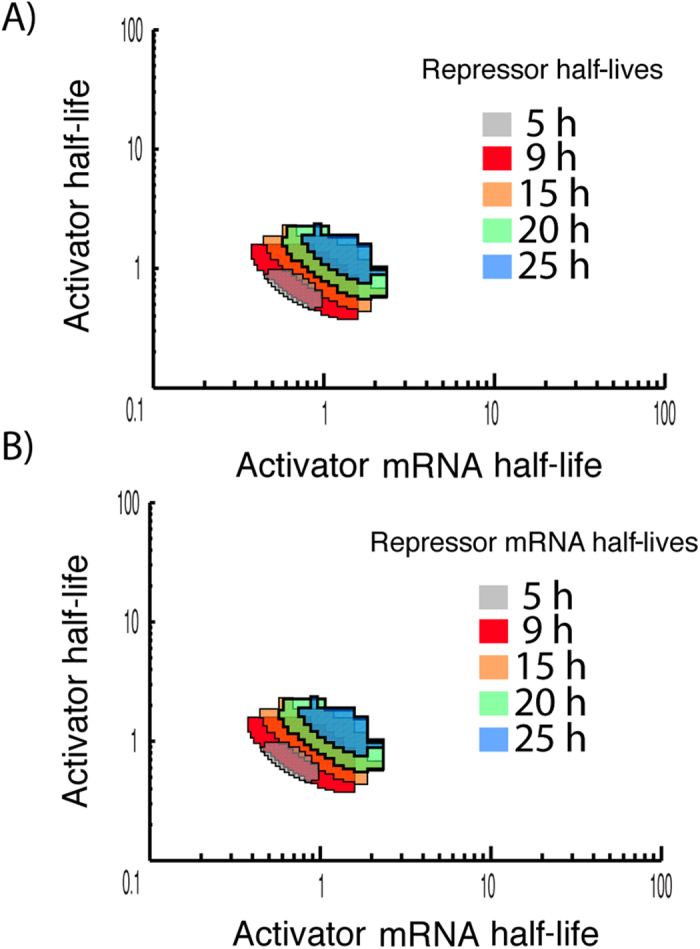
Effect of repressor mRNA and protein half-lives on oscillations. Scanning for half-lives from two hours to 35 hours shows that the system oscillates for half-lives above two hours and below 30 hours. We see that the parameters for oscillations are completely symmetrical for protein (**A**) and mRNA (**B**) half-lives. Note that the parameter range that allows oscillations is bigger for the repressor (5–25 hours) than for the activator (0.5–1.5 hours). These ranges are of biological interest, since the median of protein half-lives is 46 hours, suggesting that the system is not prone to have sustained oscillation in a biological regime.

We observe that only in an intermediate range of repressor and repressor mRNA half-lives oscillations can occur (Fig. 4A). It should be noted that the scanning range is within a biologically realistic range, i.e. half-lives from 1 to 46 hours, the lower range being rather rare (<1% of proteins)^12^. From an experimental point of view it is important to notice that if the repressor or mRNA has a half-life of 35 hours or more then oscillations can not occur, which is the case for the majority of all proteins^12^.

### Tuning the transcription rates for oscillation

**Figure 5:**
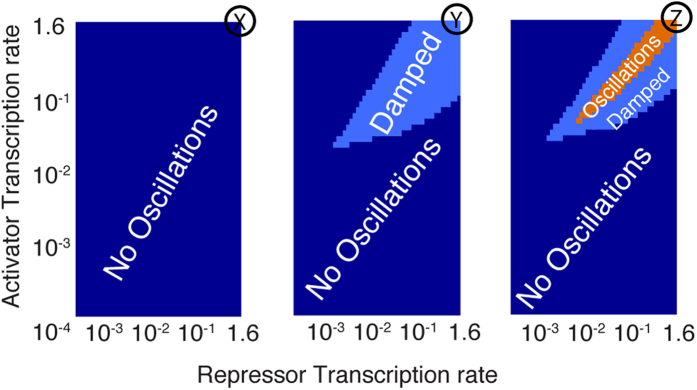
Asymmetry in transcription. The top right corner in Figure (X,Y,Z) corresponds to the parameters used for points (X,Y,Z) in Fig. 3. (**X**) Asymmetry in transcription can not produce oscillations. (**Y**) Changing the transcription asymmetry can not change damped oscillations to sustained oscillations. (**Z**) Tuning of the transcription rates is needed to produce oscillations.

The production rates for repressor and activator mRNAs (transcription rates) can be tuned by mutations in the promoter sequences. We explore the effects of decreasing the maximal transcription rate, keeping the ratios between the maximal production rates of activator and repressor mRNAs. The top right corner in Fig. 5 (X,Y,Z) corresponds to the parameters used for points (X,Y,Z) in Fig. 3. Starting from either damped oscillations (Y) or sustained oscillations (Z) we show that only a certain range of transcription rates can produce damped/sustained oscillations. Note that starting from (X) (the completely symmetric system), inducing an asymmetry in transcriptional rates does not produce oscillations. Which was the case for an asymmetry in half-lives (Fig. 3).

### The time delay has negligible effect on the function of the NAF motif

The 30 minute time delay expected from mRNA export and processing^13^,^18^ had negligible effects in the previous parameter regime for activator mRNA and protein stabilities (0.2–50 hours), with repressor and repressor mRNA half-lives fixed at 9 hours. This observation is in accordance with a previous estimate on the relation of the time delay and degradation times in transcription motifs, i.e. that the time delay (*τ*) needs to be roughly twice as long as the time scale for protein degradation 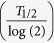
24. Therefore we preformed a wider scan, scanning mRNA and protein half-lives from 0.01 to 1000 hours for both the activator and the repressor. We contrasted the delayed and non-delayed simulations and did not find any effect of the delay (see supplementary Figure S.1). The only effect of the explicit delay for the NAF motif is the increased period of both damped and sustained oscillations by ~10%.

From a theoretical point of view it is interesting to see the effect of larger delays. We find that increasing the delay from 30 minutes actually suppresses the oscillations until a delay of 2 days where the delay induces oscillations (see supplementary Figure S.3). It should be noted that the quenching of oscillations, or amplitude death, due to a time delay has previously been explored^25^.

Since the 30 minute time delay for transcription has a negligible effect, then we suggests that for most mathematical models of mammalian transcriptional regulation motifs, using the simpler ODEs instead of the more complicated DDEs is sufficient.

### Comparison with previously explored models

**Figure 6:**
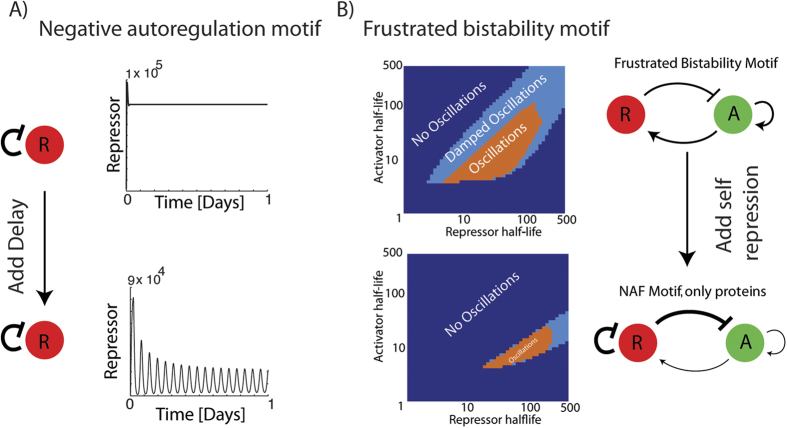
Motif comparison. (**A**) The Negative Autoregulation motif (NAR), can be recovered by setting activator transcription to zero *r*_2,*A*_ = 0. We find that when half-lives of repressor and repressor mRNA are short, a 30 minute explicit timedelay can produce oscillations. Parameters changed *r*_2,*A*_ = 0, *β*_3_ = 0.16 min^−1^, *β*_4_ = 160 min^−1^. This is qualitatively similar to dynamics of the Hes oscillator^26^. (**B**) We modified the previous frustrated bistability motif (FBM) equations to allow for autorepression, thereby creating a protein level NAF motif. When contrasting the original FBM with the protein level NAF we find that the regime with oscillations is smaller for the NAF motif. See parameters and equations in supplementary (4) and (5).

From previous reports we expect that a negative autoregulation motif (NAR) produces oscillations with unstable proteins and an explicit time delay^26^. Setting the transcription rate of activator to zero (*r*_2,*A*_ = 0), half-lives of repressor and mRNA to 4 minutes and increasing the basal transcription rate to compensate for the fast degradation of proteins (*β*_3_ = 0.16 min^−1^, *β*_4_ = 160 min^−1^), we find that an explicit time delay indeed produces oscillations (see Fig. 6). We further find that increasing the strength of the repression promotes oscillations (see supplementary Figure S.4). However when we introduce activation again (*r*_2,*A*_ = 2.3), thereby recovering the NAF motif, we find that oscillations are cancelled (see supplementary Figure S.4) This observation also holds for unstable activators and activator mRNAs (2 minute half-lives). That is, in the parameter regime where the NAR part of the NAF motif oscillates on its own, the NAF motif does not, and vice versa, when the NAF motif is allowed to oscillate the NAR can not.

It is not possible to recover the frustrated bistability motif (FBM) from our equations simply by changing parameters, as it was for the NAR motif. Instead we extend the FBM equations^6^, to include autorepression (see supplementary equations (4) and (5)). These equations take only protein levels into account. When we compare the dynamics of the NAF motif to the dynamics of the frustrated bistability motif (FBM), we find that the parameter space where the NAF motif oscillates is substantially smaller (Fig. 6B). Note that we again find that for the NAF motif to oscillate activators need to be unstable compared to repressors (Fig. 6B). We find that simply stabilizing the repressor or removing autorepression is not a guarantee for obtaining sustained oscillations; in each case there is a range of repressor-related parameter values where oscillations occur. Comparing the three motifs we can conclude that while both the NAF and the NAR motifs can produce oscillations, these motifs are less prone to oscillations than the FBM motif.

The NAF motif has been explored theoretically previously^8^,^9^,^14^,^27^, although the details of the molecular interactions are different in these models. In the simpler models, where the motif’s components are degraded at a constant rate, the NAF motif produces oscillations only when the repressor is more stable than the activator. This is in agreement with our findings. However, our analysis revealed a further requirement, i.e. that the short lived activator should be produced from an unstable mRNA. Our analysis also confirms the previous finding that the repressor’s half-life needs to be in an intermediate range for oscillations to occur^8^,^27^.

In addition to producing oscillations, a previously analyzed NAF motif was found to be bistable in certain parameter regime^8^. However, this feature seems to be sensitive to the actual genetic implementation of the circuit because a different NAF motif was found to be in a steady state, when not oscillating^27^. Similar to the latter study we did not find any bistable regime (see supplementary Figure S.2).

Finally, we note that in the somewhat different context of engineering feedback control in electronic circuits, adding a negative feedback loop to a high-gain amplifier is a well-known strategy for stabilizing and limiting the gain produced by the amplifier’s positive feedback^28^.

### The reported mRNA and protein half-lives for regulatory proteins suggest that the NAF motif is not oscillatory

**Figure 7:**
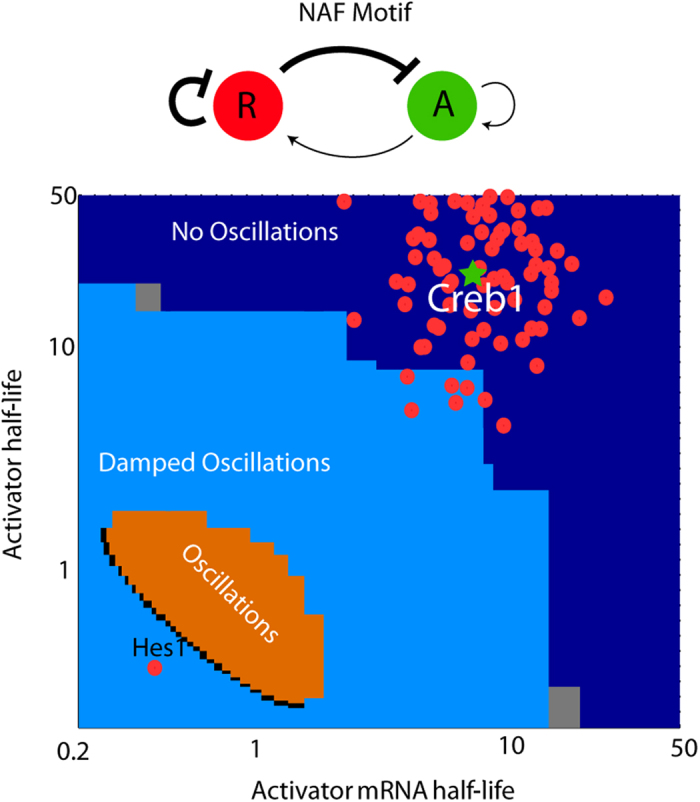
Biological regime. When plotting the half-lives for transcription factors reported by Schwanhausser *et al.*^12^ we find that almost none of them fall into the oscillatory regime. Since repressors and activators often are context dependent we do not discriminate between repressors or activators. We plot the half-life of Hes1, a negatively autoregulated repressor having oscillatory behaviour, to show that some transcription factors actually fall into the regime of (damped) oscillations^30^. However, CREB1, an activator involved in a NAF motif falls into the regime of no oscillations (green star)^8^.

We identified all transcription factors in the data set of reported protein and mRNA half-lives^12^. Overlaying the data on the half-lives of transcription factors and their corresponding mRNA with our parameter scan shows that most transcription factors fall into the non-oscillatory regime (Fig. 7). The function of transcription factors is often context dependent^29^, therefore we did not discriminate between activators and repressors. The lack of transcription factors falling into the oscillatory or even damped oscillatory regime, and the fact that the non-oscillatory regime is monostable (see supplementary figure S.2), strongly suggests that the NAF motif predominantly promotes constant protein expression.

## Conclusions

The NAF (Negative Autoregulated Frustrated bistability) motif is composed of two motifs which can promote oscillations, NAR (Negative AutoRegulated) and FBM (Frustrated Bistability Motif). This ability is retained in the NAF motif but requires features which are uncommon for mammalian regulators. The time delay in protein production due to the spatial separation of transcription and translation in eukaryotic cells has neglible effects on the dynamics of the NAF motif. In conclusion, while the NAF is capable of functioning as an oscillator, current data on transcription factors and mRNA half-lives suggest that systems governed by NAF motifs probably produce constant protein levels in mammalian cells.

## Additional Information

**How to cite this article**: Moss Bendtsen, K. *et al.* The role of mRNA and protein stability in the function of coupled positive and negative feedback systems in eukaryotic cells. *Sci. Rep.* **5**, 13910; doi: 10.1038/srep13910 (2015).

## Supplementary Material

Supplementary Information

## Figures and Tables

**Table 1:** Parameters for the model.

	Modelparameters	**Minimum**	**Median**	**Maximum**
mRNA Degradation 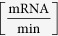 12	*γ*_1,*A*/*R*_	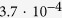	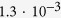	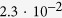
Protein Degradation 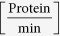 12	*γ*_2,*A*/*R*_	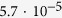	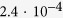	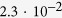
mRNA half life 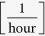 12		0.5	9	31
Protein half life 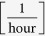 12		0.5	46	200
Translation 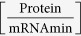 12	*r*_2_	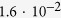	2.3	16
Transcription 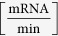 12		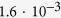	0.03	1.6
Transcription rate when activated 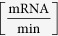 12	*β*_1_	—	1.6	—
Repressed Transcription rate 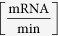	*β*_3_	—	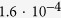	—
Unregulated Transcription rate 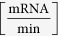 16	*β*_4_	—	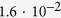	—
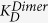 [nM]^15^	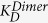	—	10	—
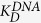 [nM]^15^	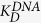	—	0.18	—

Parameters are taken from Schwanhausser *et al.* 2011^12^ and transcription rates are chosen so they mapped to the mRNA and protein abundances measured in^12^.
