# Supplementary material for: The role of mRNA and protein stability in the function of coupled positive and negative feedback systems in eukaryotic cells
Source: Sci Rep. 2015 Sep 14;5:13910. doi: 10.1038/srep13910 (PMC4568459; doi:10.1038/srep13910)
Supplement: Supplementary Information [file srep13910-s1.pdf]

The role of mRNA and protein stability in the function of coupled positive and negative feedback systems in eukaryotic cells.

Kristian Moss Bendtsen [1], Mogens H. Jensen [1a], Sandeep Krishna [1,2], Szabolcs Semsey [1]

[1] *University of Copenhagen, Niels Bohr Institute,*

Blegdamsvej 17, DK-2100 Copenhagen,

Denmark and [2] *Simons Centre for the Study of Living Machines,*

National Center for Biological Sciences,

GKVK Campus, Bellary Road, Bangalore 560065, India

[a] To whom correspondence should be addressed. Email: mhjensen@nbi.dk

SUPPLEMENTARY

Dulac's criterion

Dulac's criterion states that if we can create a real-valued function $g(\mathbf{x})$, such that $\nabla \cdot g\dot{\mathbf{x}}$ only has one sign, one can show that there can not exist a limit cycle. By choosing g=1 we get:

$$\nabla \cdot g\dot{\mathbf{x}} = \frac{\partial}{\partial P}\dot{P} + \frac{\partial}{\partial m}\dot{m} \tag{1}$$

$$= \frac{\partial}{\partial P}\left(r_2 m - \gamma_2 P\right) + \frac{\partial}{\partial m}\left(\frac{N}{ZK_{D*}^{DNA}}\left(\beta_1^* P P_{tot}^* + \beta_2^* P P_{tot}^* + \beta_3^* P P_{tot}^*\right) + N\beta_4^* - \gamma_1 m\right) \tag{2}$$

$$= -\gamma_2 - \gamma_1 \tag{3}$$

Since the degradation rates are always positive, the divergence is always negative, hence there cannot exits a limit cycle in the two-dimensional case, which means no oscillations in the completely symmetric case. Since this only relies on positive values of the rates, we can state that no matter which parameter values we chose, the completely symmetrical case will never be able to produce oscillations.

Half-lives parameter scans: 0.01 - 1000 hours

To limit computation time and keep a reasonable fine grained scan, we choose to scan parameters in different regimes. In total we performed 144 parameter scans, each scanning 50x50 grid parameter space for the activator and activator mRNA half-lifes. We keep repressor and repressor mRNA at a value equal to the half of the upper limit of the studied parameter range. The means that the parameter range (activator fast, activator mRNA fast, repressor fast, repressor mRNA fast) is scanning activator and activator mRNA half-lives from 0.01 to 1 hour while keeping repressor and repressor mRNA half-lives at 0.5 hour.
The half-lives ranges are listed in the table:

PARAMETER RANGES:	Lower limit [hours]	Upper limit [hours]
Fast mRNA/Protein	0.01	1
Medium mRNA/ Comparable Protein	1	25
Slow mRNA/Medium Protein	25	100
Slow Protein	100	1000

TABLE I. Half-life parameter ranges.

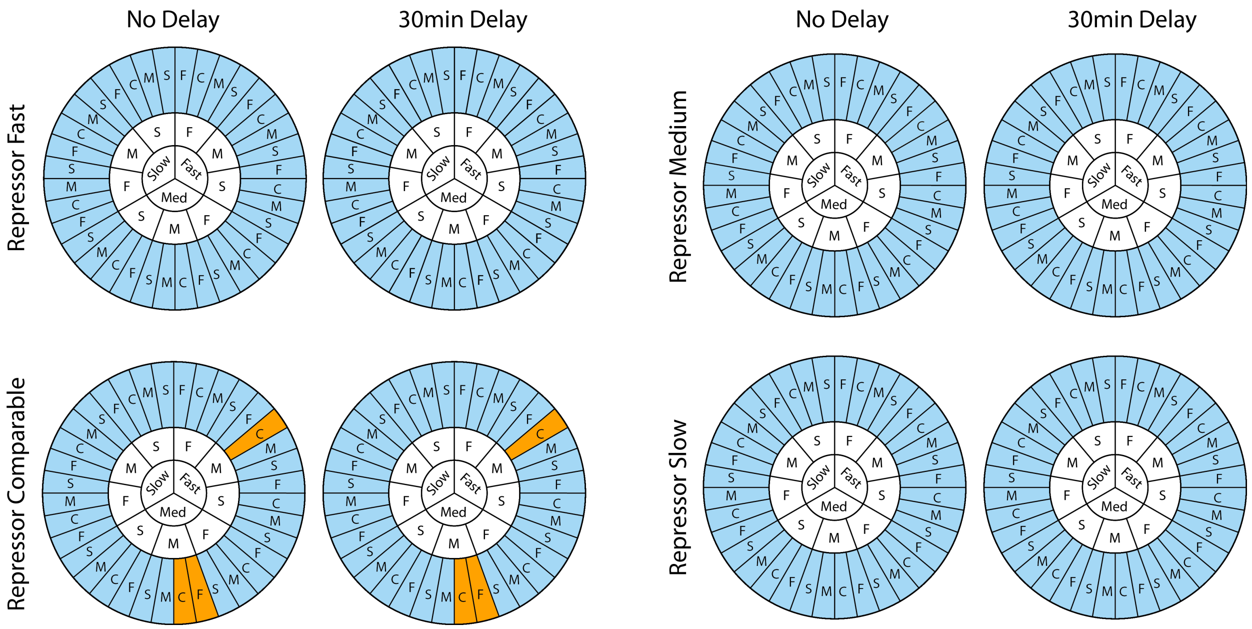

FIG. 1. **Half-life parameter scans in the interval 0.01 - 1000 hours:**

Scanning the half lives shows that areas with oscillations (orange) and the regimes with on oscillations (blue) are the same for no delay and for 30min time delays. The innermost ring is the activator mRNA half-life, the middle ring is the repressor mRNA half-life and the outer most ring is the activator half-life.

Frustrated bistability motif and simplified NAF equations:

As mentioned in the paper, we simplify the NAF equations by only taking protein levels as dynamical variables:

$$\frac{dA}{dt} = \alpha \frac{1}{1+\frac{R}{K}^2} \frac{b+\frac{A}{K}^2}{1+\frac{A}{K}^2} - \gamma_1 A \tag{4}$$

$$\frac{dR}{dt} = \alpha \overbrace{\frac{1}{1+\frac{R}{K}^2}}^{\text{NAR}} \frac{b+\frac{A}{K}^2}{1+\frac{A}{K}^2} - \gamma_2 R \tag{5}$$

Here A is the activator, R is the repressor, b=0.0016 is leakages of the promoter, α=2.3 is the translation rate, K=55 is the ratio of dimerization dissociation constant to DNA dissociation constant, and γ is the degradation rates. The NAR term (indicated by the curly bracket) is set equal to 1 for the frustrated bistability motif. The equations can easily be made dimensionless, but for our purpose of comparing directly with biological parameters, it is not useful first to make the quantities dimensionless.

System is monostable in non-oscillatory regime

We find two fixed points of the system, analyzing the stability of these we find that the system is monostable in the non-oscillatory regime, see figure 2.

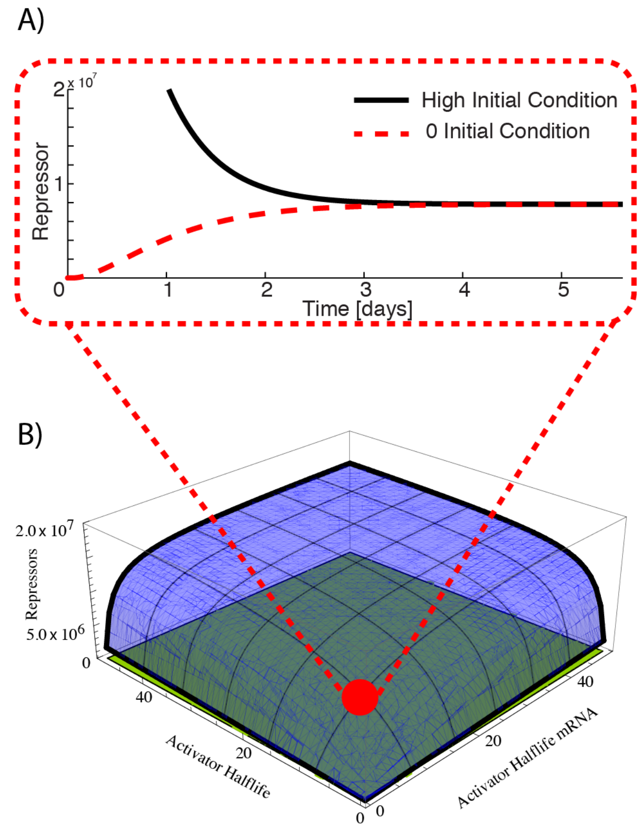

FIG. 2. **The non-oscillatory regime is monostable:**

We plot the fixed points for the repressor within the same regime as scanned in figure 3. **B)** The system has two solutions, the yellow and the blue. **A)** Testing for stability, we see that only the blue solution is stable.

Effect of large time delays

We show that increasing the time delay supresses the oscillations until a delay of 2 day where they reappear see figure 3.

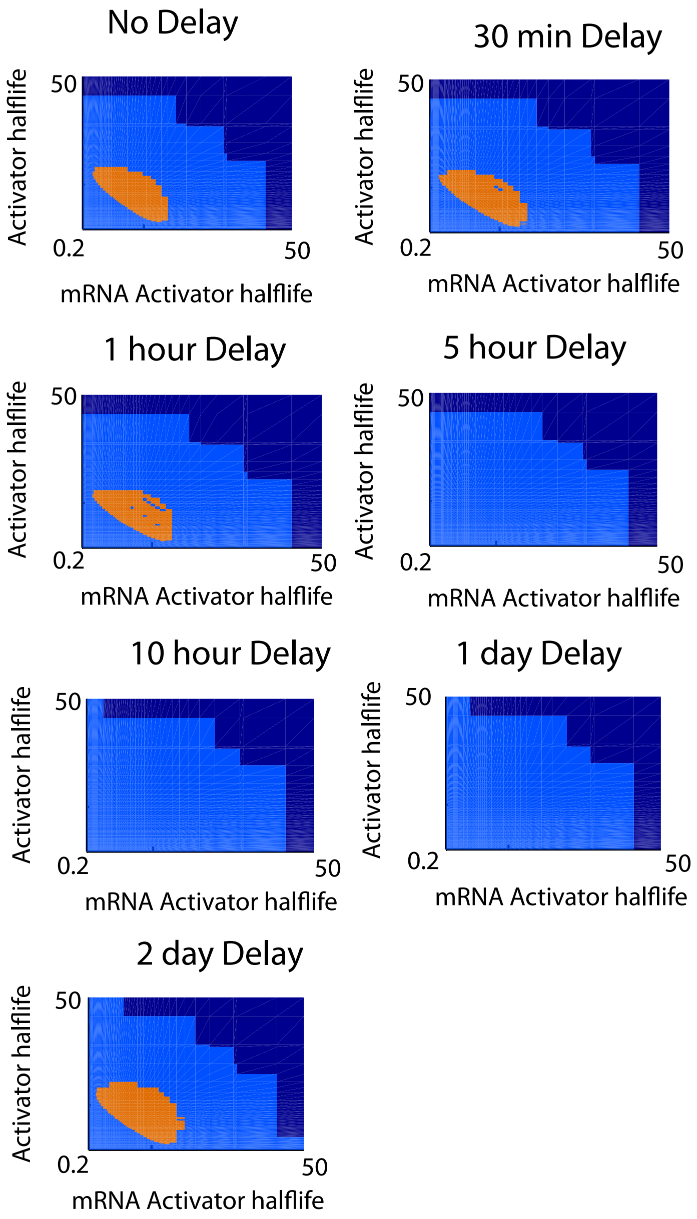

FIG. 3. **Effect of time delays**

Even though we show that a biological realistic time delay of 30 minutes has no effect on our system, it is theoretically interesting to see what effect larger time delays will have. We scanned the time delays for (0, 0.5 hours, 1 hour, 5 hours, 10 hours, 1 day and 2 days), and surprisingly we found that increasing the time delay actually quenched the oscillations when the time delays are between 5 hours and 1 day. For a time delay of 2 days, the oscillations were again induced in a small parameter range. Because the amplitudes of the 2 day delay oscillations were less than 2 fold we used the damping coefficient criteria to classify the regimes.

Classifying dynamical behaviour of NAF model

We distinguish between three types of dynamics: Sustained oscillations, damped Oscillations and no oscillations. We used a modifying version of the matlab function *findpeaks*, to find peaks in the time series of the repressor. To ensure that peaks originating from numerical errors were discarded, we only included peaks with atleast a 2 fold amplitude. Simulations were run such that the length of each time series corresponded to 50 days. The dynamics were classified as follows:

- **Sustained oscillations:** 10 or more oscillations
- **Damped oscillations:** 9 or fewer oscillations
- **No Oscillations** No peaks

There is agreement between this classification and the linear stability of the fixed points (Compare figure 3 A,C.) The fixed points for the linear stability analysis were numerically computed using Mathematica.

Perturbation of the motif

For very unstable repressors and repressor mRNA (half-lives of 4 minutes), we find that a 30 minute delay can induce oscillations, and with addition of activators even with short half-lives (4 minutes), it supresses the oscillations, see figure 4.

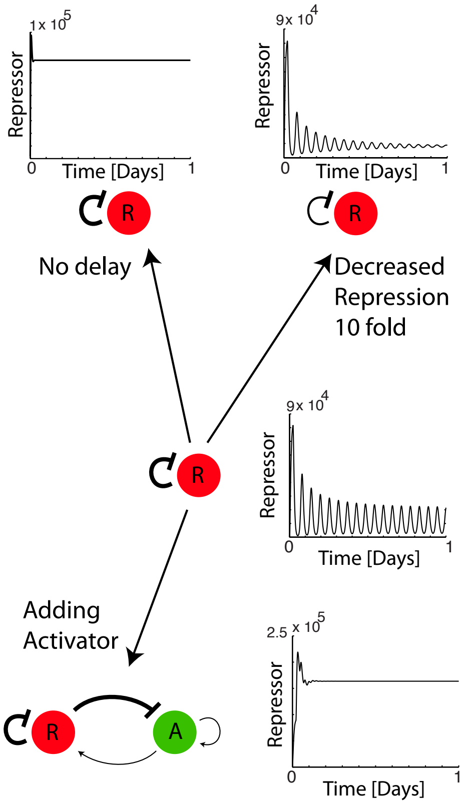

FIG. 4. **Motif Perturbations**

For mRNA and proteins half-lives of 4 minutes, we show that a 30 min time delay can induce oscillations. We also show that decreasing the repressing strength removes oscillations. Surprisingly the addition of an activator with a low transcription rate with half-lives of 4 minutes removes the oscillations. The oscillations can be recovered if the logic of regulations is such that a heteromer (activator-repressor) also represses. Base parameters used for NAR motif is: $r_{2,R} = 2.3\ \mathrm{min}^{-1}$, $r_{2,A} = 0\ \mathrm{min}^{-1}$, $\beta_1 = 1.6\ \mathrm{min}^{-1}$, $\beta_2 = 0.8\ \mathrm{min}^{-1}$,$\beta_3 = 1.6 \cdot 10^{-1}\mathrm{min}^{-1}$,$\beta_4 = 1.6 \cdot 10^{2}\mathrm{min}^{-1}$,$K_D^{Dimer} = 10$ nM,$K_D^{DNA} = 0.18$ nM, $\tau = 30$min.

Removing the delay corresponds to $\tau = 30$min. Decrease repression correspond to $\beta_3 = 1.6\mathrm{min}^{-1}$. Adding an activator correspond to $r_{2,A} = 2.3\ \mathrm{min}^{-1}$, and $\beta_1 = 1.6\ \mathrm{min}^{-1} \cdot 10^3$, $\beta_2 = 0.8\ \mathrm{min}^{-1} \cdot 10^3$,$\beta_3 = 1.6 \cdot 10^{-1}\mathrm{min}^{-1}$,$\beta_4 = 1.6 \cdot 10^{2}\mathrm{min}^{-1}$ The degradation rates of the repressor mRNA and repressor is: $\gamma_{(1/2,R)} = 0.17\mathrm{min}^{-1}$ and activator and activator mRNA $\gamma_{(1/2,R)} = 0.34\mathrm{min}^{-1}$

Transcription rate $\langle r_1(\tau)\rangle_{(A,R)}$:

$$\langle r_1(\tau)\rangle_{(A,R)} = \frac{N}{ZK_{D*}^{DNA}}\left(\beta_1^* AA_{tot}^* + \beta_2^* AR_{tot}^* + \beta_3^* RR_{tot}^*\right) + N\beta_4^* \tag{6}$$

here AA_{tot}^* is the total number of dimer-activators, RR_{tot}^* is the total of dimer-repressors,AR_{tot}^* is the total of hetero-dimers, K_{D*}^{DNA} is the dimensionless dissociation for the protein-DNA binding. β_1 is activated transcription rate, β_2 is heterodimer transcription rate, β_3 is repression transcription rate and β_4 is unregulated(basal) transcription. N is the number of regulator sites.

Deriving the transcription rate $\langle r_1(\tau)\rangle_{(A,R)}$:

Dimerization

We have the following equation for dimerization of TetR and TetA:

$$\frac{d\,[RR]}{dt} = k_{on}^1\,[R]\,[R] - k_{off}^1\,[RR] \tag{7}$$

$$\frac{d\,[AR]}{dt} = k_{on}^2\,[A]\,[R] - k_{off}^2\,[AR] \tag{8}$$

$$\frac{d\,[AA]}{dt} = k_{on}^3\,[A]\,[A] - k_{off}^3\,[AA] \tag{9}$$

Due to biochemical identities we have that: $k_{on} = k_{on}^1 = k_{on}^2 = k_{on}^3$ and $k_{off} = k_{off}^1 = k_{off}^2 = k_{off}^3$. Solving for steady state we get that:

$$[R]^2 = K_D^{Dimer}\,[RR] \tag{10}$$

$$[A]^2 = K_D^{Dimer}\,[AA] \tag{11}$$

$$[A]\,[R] = K_D^{Dimer}\,[AR] \tag{12}$$

In addition we have that the total activator and repressor concentration is conserved on short timescales:

$$[A]_{tot} = [A] + 2\,[AA]_{tot} + [AR]_{tot} = [A] + 2\,[AA] + 2\,[AAO] + [AR] + [ARO] \tag{13}$$

$$[R]_{tot} = [R] + 2\,[RR]_{tot} + [AR]_{tot} = [R] + 2\,[RR] + 2\,[RRO] + [AR] + [ARO] \tag{14}$$

Where $[AAO]$ is the amount of activators (or repressors) bound to the operator sites. Since the number of operator sites is small (roughly 7), we assume that the majority of dimers are

free. $[TF]_{tot} \approx [TF]$.

We then get:

$$[A]_{tot} = [A] + 2\,[AA]_{tot} + [AR]_{tot} \tag{15}$$

$$[R]_{tot} = [R] + 2\,[RR]_{tot} + [AR]_{tot} \tag{16}$$

and

$$[R]^2 = K_D^{Dimer}\,[RR]_{tot} \tag{17}$$

$$[A]^2 = K_D^{Dimer}\,[AA]_{tot} \tag{18}$$

$$[A]\,[R] = K_D^{Dimer}\,[AR]_{tot} \tag{19}$$

We are interested is an algebraic relation which gives us the free dimer concentrations when we consider the total concentrations:

$$[A] + 2\frac{[A]^2}{K_D^{Dimer}} + \frac{[A]\,[R]}{K_D^{Dimer}} - [A]_{tot} = 0 \tag{20}$$

$$[R] + 2\frac{[R]^2}{K_D^{Dimer}} + \frac{[A]\,[R]}{K_D^{Dimer}} - [R]_{tot} = 0 \tag{21}$$

Solving the first equation with respect to $[A]$, we obtain:

$$[A] = \frac{1}{4}\left(\sqrt{8 \cdot [A]_{tot} \cdot K_D^{Dimer} + K_D^{Dimer^2} + 2K_D^{Dimer}\,[R] + [R]^2} - K_D^{Dimer} - [R]\right) \tag{22}$$

Inserting this into the second equation:

$$\frac{1}{4}\left(\sqrt{8 \cdot [A]_{tot} \cdot K_D^{Dimer} + K_D^{Dimer^2} + 2K_D^{Dimer}\,[R] + [R]^2} - K_D^{Dimer} - [R]\right)\frac{[R]}{K_D^{Dimer}} + \tag{23}$$

$$[R] + 2\frac{[R]^2}{K_D^{Dimer}} - [R]_{tot} = 0 \tag{24}$$

The solution to this equation is so cumbersome that it is easier to solve numerical for each time step using the Newton method with tolerance of 10^{-3}.

For the later equations we denote the solutions for R and A as A^* and R^*, and the dimers as:

$$AA^*_{tot} = \frac{A^{*2}}{K_D^{Dimer}} \tag{25}$$

$$RR^*_{tot} = \frac{R^{*2}}{K_D^{Dimer}} \tag{26}$$

$$AR^*_{tot} = \frac{A^*R^*}{K_D^{Dimer}} \tag{27}$$

The concentration equations can be changed to equations for protein numbers by changing the dissociation so they are dimensionless (by multiplying with the nucleus volume and Avogadro's number). $K_D^{Dimer} \cdot V_{nucleus} \cdot C_A = K_{D*}^{Dimer}$. For HeLa cells the nuclear volume has been measured to almost double during cell cycle, from roughly 90010^{-18} m^3 to almost 2000 [1], we use $1650 \cdot 10^{-18}$ m^3 as a rough average for mammalian cells.

DNA binding

In addition to the dimerization the dimers bind to the operator sites on the DNA.

The total concentration for a single operator site is the sum of free and bound operator site.

$$[O]_{tot} = [O]_{free} + [RRO] + [ARO] + [AAO] \tag{28}$$

The differential equation governing a single operator site binding is:

$$\frac{d\,[AAO]}{dt} = k_{on}^{DNA}\,[AA]\,[O] - k_{off}^{DNA}\,[AAO] \tag{29}$$

$$\frac{d\,[ARO]}{dt} = k_{on}^{DNA}\,[AR]\,[O] - k_{off}^{DNA}\,[ARO] \tag{30}$$

$$\frac{d\,[RRO]}{dt} = k_{on}^{DNA}\,[RR]\,[O] - k_{off}^{DNA}\,[RRO] \tag{31}$$

Note, that the biochemical "symmetries" mean that every dimer interacts in the same way with the DNA and the on rates and the off rates are therefore equal for dimers. The total transcription factor concentration is both the concentration of transcription factor bound to the operator site and free transcription factor.

$$[RR]_{tot} = [RR] + [RRO] \tag{32}$$

$$[AR]_{tot} = [AR] + [ARO] \tag{33}$$

$$[AA]_{tot} = [AA] + [AAO] \tag{34}$$

However we previously assumed that the bound fraction is small compared to the free fraction. Which means that the free concentration is almost the total concentration, $[TF]_{tot} \approx$

$[TF]$. Using this assumption, solving the DNA binding for steady state we find that:

$$[AAO] = \frac{[AA]_{tot}}{K_D^{DNA}}[O] = \frac{AA_{tot}}{K_{D*}^{DNA}}[O] \tag{35}$$

$$[ARO] = \frac{[AR]_{tot}}{K_D^{DNA}}[O] = \frac{AR_{tot}}{K_{D*}^{DNA}}[O] \tag{36}$$

$$[RRO] = \frac{[RR]_{tot}}{K_D^{DNA}}[O] = \frac{RR_{tot}}{K_{D*}^{DNA}}[O] \tag{37}$$

Where the dissociation constants are made dimensionless

Using the steady states and conservation of operator sites we can calculate the probability for a single promoter to be free, bound by activator dimers AA, activator-repressor dimers AR, or repressor dimers RR.

$$[O]_{tot} = [O] + \left(\frac{AA_{tot}}{K_{D*}^{DNA}} + \frac{RR_{tot}}{K_{D*}^{DNA}} + \frac{AR_{tot}}{K_{D*}^{DNA}}\right)[O] \tag{38}$$

$$P(free) = \frac{[O]}{[O]_{tot}} = \frac{1}{1 + \frac{AA_{tot}^*}{K_{D*}^{DNA}} + \frac{RR_{tot}^*}{K_{D*}^{DNA}} + \frac{AR_{tot}^*}{K_{D*}^{DNA}}} = \frac{1}{Z} \tag{39}$$

$$P(AA) = \frac{[AAO]}{[O]_{tot}} = \frac{AA_{tot}^*}{K_{D*}^{DNA}}\frac{1}{1 + \frac{AA_{tot}^*}{K_{D*}^{DNA}} + \frac{RR_{tot}^*}{K_{D*}^{DNA}} + \frac{AR_{tot}^*}{K_{D*}^{DNA}}} = \frac{AA_{tot}^*}{K_{D*}^{DNA}}\frac{1}{Z} \tag{40}$$

$$P(AR) = \frac{[ARO]}{[O]_{tot}} = \frac{AR_{tot}^*}{K_{D*}^{DNA}}\frac{1}{1 + \frac{AA_{tot}^*}{K_{D*}^{DNA}} + \frac{RR_{tot}^*}{K_{D*}^{DNA}} + \frac{AR_{tot}^*}{K_{D*}^{DNA}}} = \frac{AR_{tot}^*}{K_{D*}^{DNA}}\frac{1}{Z} \tag{41}$$

$$P(RR) = \frac{[RRO]}{[O]_{tot}} = \frac{RR_{tot}^*}{K_{D*}^{DNA}}\frac{1}{1 + \frac{AA_{tot}^*}{K_{D*}^{DNA}} + \frac{RR_{tot}^*}{K_{D*}^{DNA}} + \frac{AR_{tot}^*}{K_{D*}^{DNA}}} = \frac{RR_{tot}^*}{K_{D*}^{DNA}}\frac{1}{Z} \tag{42}$$

Transcriptional probability

From the probabilities for the states of the operator sites we can calculate the probabilities of transcription, which for a single operator site gives the rate of transcription as:

$$r_1 = \beta_1 P(AA) + \beta_2 P(AR) + \beta_3 P(RR) + \beta_4 P(free) \tag{43}$$

We assume there is a linear relation between occupancy and transcription probability, so for N operator sites the average rate is given as:

$$\langle r_1 \rangle = \beta_1 \langle n_{AA} \rangle + \beta_2 \langle n_{AR} \rangle + \beta_3 \langle n_{RR} \rangle + \beta_4 \langle free \rangle \tag{44}$$

We have that $\langle n_{AA} \rangle = N \cdot P(AA)$ where again N is the number of operator sites and P(AA) is the probability the activation dimer has bound. Since $P(free) = 1 - P(AA) - P(AR) -$

$P(RR)$ we get that

$$\langle r_1\rangle = (\beta_1 - \beta_4)\langle n_{AA}\rangle + (\beta_2 - \beta_4)\langle n_{AR}\rangle + (\beta_3 - \beta_4)\langle n_{RR}\rangle + \beta_4 N \tag{45}$$

$$\tag{46}$$

$$\langle r_1\rangle = N\beta_1^* P(AA) + N\beta_2^* P(AR) + N\beta_3^* P(RR) + N\beta_4 \tag{47}$$

This can be written using the total number of dimer proteins AA^*_{tot}, AR^*_{tot} and RR^*_{tot}. The transcription rates are related to is other in the following way. $\beta_1 > \beta_2 > \beta_4 > 0 > \beta_3$. Furthermore the heterodimer activation is simply the average between the activation and repression $\beta_2 = \frac{\beta_1+\beta_3}{2}$.

$$\langle r_1\rangle = \frac{N}{ZK^{DNA}_{D*}}\left(\beta_1^* AA^*_{tot} + \beta_2^* AR^*_{tot} + \beta_3^* RR^*_{tot}\right) + N\beta_4 \tag{48}$$

Where AA^*_{tot}, AR^*_{tot} and RR^*_{tot} are numerically calculated from the total concentration of activator and repressor A_{tot} and R_{tot}

$$\dot{A_{tot}} = r_2 mRNA_A - \gamma_2 A_{tot} \tag{49}$$

$$\dot{R_{tot}} = r_2 mRNA_R - \gamma_2 R_{tot} \tag{50}$$

$$m\dot{RNA}_A = \langle r_1\rangle - \gamma_1 mRNA_A \tag{51}$$

$$m\dot{RNA}_R = \langle r_1\rangle - \gamma_1 mRNA_R \tag{52}$$

[1] Maeshima, K. *et al.* Nuclear pore formation but not nuclear growth is governed by cyclin-dependent kinases (cdks) during interphase. *Nature structural & molecular biology* **17**, 1065–1071 (2010).
